# Association Between Preoperative Dyslipidemia and the Prognosis of Patients With Endometrial Cancer: A Retrospective Cohort Study

**DOI:** 10.1002/kjm2.70195

**Published:** 2026-03-09

**Authors:** Song Li, Rong‐He Sun, Qing Chen, Zeng‐Shu Huang, Yun‐Fei Wang, Chang‐Zhong Li

**Affiliations:** ^1^ Center of Obstetrics and Gynecology Peking University Shenzhen Hospital, Shenzhen Peking University‐The Hong Kong University of Science and Technology Medical Center Shenzhen Guangdong China; ^2^ Center of Reproductive Medicine, Shenzhen People's Hospital Shenzhen Guangdong China; ^3^ Affiliated Hospital of Jining Medical University, Jining Medical University Clinical College Jining Shandong China; ^4^ Institute of Obstetrics and Gynecology, Shenzhen PKU‐HKUST Medical Center Shenzhen Guangdong China; ^5^ Shenzhen Key Laboratory on Technology for Early Diagnosis of Major Gynecologic Diseases Shenzhen Guangdong China

**Keywords:** dyslipidemia, endometrial cancer, overall survival, prognosis

## Abstract

Endometrial cancer (EC) is closely related to metabolic disorders. We aimed to investigate whether preoperative dyslipidemia independently affects the prognosis of patients with EC. One hundred and ninety‐four women diagnosed with EC by pathology and who had undergone standardized surgical treatment at the Affiliated Hospital of Jining Medical College between June 2013 and December 2016 were enrolled. Participants were divided into high and low blood lipid groups based on the appropriate and abnormal stratification criteria in the Chinese Blood Lipid Management Guidelines (2023). Their clinical characteristics, preoperative blood lipids, and survival data were retrospectively collected and analyzed. A univariate Cox proportional hazards regression model showed that age at diagnosis, preoperative triglycerides (TG), preoperative high‐density lipoprotein cholesterol (HDL‐C), International Federation of Obstetrics and Gynecology (FIGO) stage, and histological type were risk factors affecting overall survival (OS) in patients with EC (*p* < 0.05). Furthermore, preoperative high TG levels (≥ 1.7 mmol/L) and low HDL‐C levels (< 1.0 mmol/L) had statistically significant negative impacts on the OS of patients with EC (both *p* < 0.05) in a multivariate Cox regression model. Moreover, the median survival time significantly differed based on TG and HDL‐C levels (low‐level vs high‐level: 91.2 months vs 81.6 months, *p* < 0.05; 72.8 months vs. 90.9 months, *p* < 0.05; respectively). In conclusion, higher TG or lower HDL‐C levels are associated with worse prognoses in patients with EC, which could serve as important biomarkers for evaluating the prognosis of patients with EC.

## 1 Introduction

Endometrial cancer (EC) is one of the most common gynecological malignancies in China [1], with its incidence and mortality rates increasing every year, with a trend toward an increasing number of cases in younger women [2]. According to data released by the National Cancer Center in 2022 [3], the incidence rate of EC in China is 10.54 per 100,000, while the mortality rate is 2.53 per 100,000. In recent years, the incidence rate of EC in China has increased significantly due to improvements in living standards and lifestyle changes, including higher fat and sugar consumption, as well as reduced exercise [4]. The prognosis of patients with EC mainly depends on the patient's age at diagnosis, the pathological stage and type, and the degree of tumor differentiation. Most patients are easily diagnosed in the early stages, and precise surgical intervention in the early stages can result in a good 5‐year survival rate of up to 95% [5]. However, the 5‐year survival rate for patients with local spread and distant metastasis outside the uterus is poor, being 68% and 17%, respectively [6]. Radiotherapy, hormonal therapy, and chemotherapy have been used as adjuvant treatments for EC, but these treatment methods have certain limitations. Therefore, finding accurate, effective, and inexpensive pretreatment biomarkers for evaluating the prognosis of EC is very important.

The impact of metabolic disorders on carcinogenesis and cancer progression has been widely discussed. Lipids are the general term for plasma neutral fats and lipids, mainly including triglycerides (TG), total cholesterol (TC), high‐density lipoprotein cholesterol (HDL‐C), and low‐density lipoprotein cholesterol (LDL‐C). In recent years, the incidence of dyslipidemia in Chinese adults has remained at a relatively high level [7]. Abnormal blood lipids not only increase the risk of cardiovascular disease but also contribute to tumor development, which might lead to a poor prognosis among patients with malignancies. Studies have shown that dyslipidemia can promote the occurrence and development of breast cancer, colorectal cancer, and other malignant tumors [8, 9, 10, 11], and there are lipid‐regulating drugs for the adjuvant treatment of patients with tumors [12, 13, 14]. However, the interaction between abnormal blood lipid metabolism and the tumor microenvironment is intricate and largely unknown, and there are few studies on the relationship between blood lipid levels and the prognosis of patients with EC. The previous research that reported the association between dyslipidemia and EC risk and prognosis had not excluded those patients with chronic diseases such as hypertension, diabetes and coronary heart disease which might affect blood lipid metabolism. A study by Luo et al. reported an association between the pretreatment TG‐to‐HDL‐C (TG/HDL‐C) ratio and EC in postmenopausal women; this was a case–control study that did not exclude patients with hypertension and diabetes mellitus [15]. They also reported the relationship between the TG/HDL‐C ratio and the clinicopathological characteristics of 167 patients with EC; however, they did not report the survival of these patients. As EC is closely related to metabolic disorders, understanding how dyslipidemia influences the prognosis of patients with EC may be valuable, and dyslipidemia may become a new target for anti‐cancer treatment in the future.

In the present study, we retrospectively analyzed the association between the preoperative blood lipid levels and the pathological characteristics of EC, as well as the overall survival (OS) of patients with EC. We aimed to clarify the relationship between preoperative blood lipid levels and the OS of patients with EC, and investigate whether preoperative dyslipidemia could serve important biomarkers for evaluating the prognosis of patients with EC, thus providing a new perspective for the diagnosis and treatment of EC.

## 2 Materials and Methods

### 2.1 Participants

**FIGURE 1:**
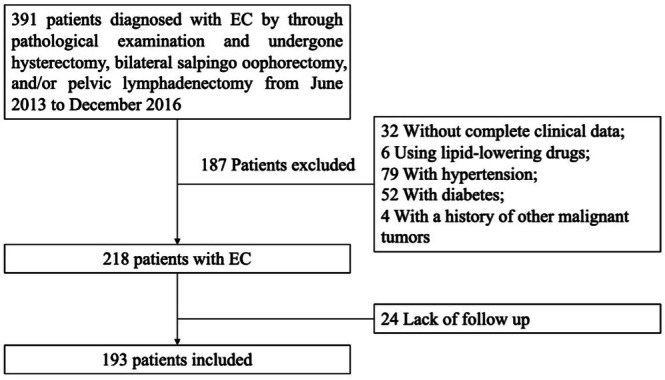
Flowchart of the cohort. EC: endometrial cancer.

We included 391 patients who had been diagnosed with EC by pathological examination and undergone hysterectomy, bilateral salpingo‐oophorectomy, and/or pelvic lymphadenectomy in the Affiliated Hospital of Jining Medical College between June 2013 and December 2016. The exclusion criteria were as follows: (1) Patients without follow‐up or complete clinical data; (2) Patients with chronic diseases, such as hypertension, diabetes, and coronary heart disease, that may affect blood lipid metabolism; (3) Patients with a history of other malignant tumors; and (4) Patients who received chemotherapy, radiotherapy, and other treatments before surgery. One hundred and ninety‐four patients were ultimately enrolled. We then collected data, including patients' age at surgery, body mass index (BMI), menopausal status, blood lipid measurement values within 1 week before surgery, International Federation of Obstetrics and Gynecology (FIGO) stage, differentiation grading, pathological type, tumor size, lymph node metastasis status, and myometrial invasion depth. The inclusion and exclusion details of the analyzed cohort are shown in Figure 1.

All patients had been evaluated and operated upon by experienced gynecologists in the Affiliated Hospital of Jining Medical College. Experienced pathologists carefully reviewed the surgical specimens to determine the diagnosis, pathological type, histological subtype, and myometrial invasion depth. EC was staged according to the 2009 FIGO guidelines for EC. This study was performed in line with the principles of the Declaration of Helsinki. The Research Ethics Committee of the Affiliated Hospital of Jining Medical University approved the study and waived the need for informed consent.

### 2.2 Determination and Grouping of Blood Lipids

Fasting blood samples were collected, and lipids (TC, TG, HDL‐C, and LDL‐C) were measured using a Cobas 8000 c702 fully automatic biochemical immunoassay analyzer. We grouped patients according to the appropriate level and abnormal stratification criteria for blood lipids in the 2023 Chinese guideline for lipid management [16], with a cutoff value of 5.2 mmol/L for TC, 1.7 mmol/L for TG, 1.0 mmol/L for HDL‐C, and 3.4 mmol/L for LDL‐C. Patients were grouped into the low‐level group if the measured value was less than the cutoff value; otherwise, they were included in the high‐level group.

### 2.3 Follow‐Up

Patient follow‐up was conducted through telephone calls and the medical record system. Follow‐up was performed in December 2021. The endpoint of this study was death or the end of follow‐up. Survival time was defined as the overall survival time, which was the time from surgery to death (measured in months).

### 2.4 Statistical Analysis

Data are presented as the mean ± SD or median (range) for continuous variables and as *n* (%) for categorical variables. The differences in continuous variables between two groups were analyzed using an independent‐sample *t*‐test if the data followed the normal distribution and the variances between the two groups were equal; otherwise, Mann–Whitney *U* tests were applied. The chi‐square test was used to compare categorical variables between groups. The univariate Cox regression analysis method was used to identify variables significantly associated with OS (*p* < 0.05), which were then included in the multivariate Cox regression model. For the multi‐categorical variables, appropriate reference layers were selected to establish the multivariate Cox regression model, and the hazard ratio (HR) was used as the risk assessment parameter. Based on a Kaplan–Meier analysis, a log rank test was used to compare different levels, and survival curves were plotted. SPSS version 26.0 was used for data analysis. A *p* value of less than 0.05 indicated statistical significance.

## 3 Results

The baseline demographic and clinical characteristics of the included patients (*n* = 194) are presented in Table S1. The median age at EC diagnosis was 52.9 years (IQR: 50–59). Over 52% of the cases were postmenopausal. Most of the 194 (82.0%) patients had a pathological diagnosis of endometrioid EC. Furthermore, 156 (80.4%) patients were diagnosed with FIGO stage I disease, while 38 (19.6%) were diagnosed with FIGO stage II or III disease. The tumor grade was G1–G2 in 158 (81.4%) cases and G3 in 36 (18.6%) cases. Most cases had negative cervical stromal involvement (83.5%), < 50% myometrial invasion (73.7%), and negative lymph node metastasis (86.1%).

The clinical and pathological characteristics of patients with EC in the low‐level and high‐level lipid groups are shown in Table 1. The average age, menopausal status, and BMI were not significantly different between the low‐level and high‐level lipid groups. In the high‐level TC group, the proportion of patients with non‐endometrioid adenocarcinoma was higher. The proportion of patients with lymph node metastasis was higher in the high‐level than in the low‐level TG group. In the low‐level HDL‐C group, more patients had a FIGO stage of more than I or underwent postoperative adjuvant therapy. In the high‐level LDL‐C group, more patients had non‐endometrioid adenocarcinoma, the largest diameter of the tumor ≥ 2 cm, myometrial invasion depth greater than or equal to half, and postoperative adjuvant therapy.

**TABLE 1:** clinical characteristics in patients with EC of low‐level and high‐level lipids groups, *n* (%).

		TC		*p*	TG		*p*	HDL‐C		*p*	LDL‐C		*p*
		< 5.2 mmol/L	≥ 5.2 mmol/L		< 1.7 mmol/L	≥ 1.7 mmol/L		< 1.0 mmol/L	≥ 1.0 mmol/L		< 3.4 mmol/L	≥ 3.4 mmol/L	
*n*		128	66		132	62		40	154		150	44	0.283
Age (years)	< 55	80 (62.5%)	41 (62.1%)	0.959	85 (64.4%)	36 (58.1%)	0.396	25 (62.5%)	96 (62.3%)	0.985	96 (64.0%)	25 (56.8%)	0.387
	≥ 55	48 (37.5%)	25 (37.9%)		47 (35.6%)	26 (41.9%)		15 (37.5%)	58 (37.7%)		54 (36.0%)	19 (43.2%)	
Menopause status	Premenopausal	66 (51.6%)	27 (40.9%)	0.159	67 (50.8%)	26 (41.9%)	0.251	14 (35%)	79 (51.3%)	0.066	76 (50.7%)	17 (38.6%)	0.16
	Postmenopausal	62 (48.4%)	39 (59.1%)		65 (49.2%)	36 (58.1%)		26 (65%)	75 (48.7%)		74 (49.3%)	27 (61.4%)	
BMI (kg/m^2^)	< 25	60 (46.9%)	32 (48.5%)	0.832	66 (50.0%)	26 (41.9%)	0.294	16 (40.0%)	76 (49.4%)	0.291	71 (47.3%)	21 (47.7%)	0.963
	≥ 25	68 (53.1%)	34 (51.5%)		66 (50.0%)	36 (58.1%)		24 (60.0%)	78 (50.6%)		79 (52.7%)	23 (52.3%)	
FIGO stage	I	106 (82.8%)	50 (75.8%)	0.241	106 (80.3%)	50 (80.6%)	0.955	27 (67.5%)	129 (83.8%)	0.021	122 (81.3%)	34 (77.3%)	0.551
	> I (II + III)	22 (17.2%)	16 (24.2%)		26 (19.7%)	12 (19.4%)		13 (32.%5)	25 (16.2%)		28 (18.7%)	10 (22.7%)	
Pathological type	Endometrioid adenocarcinoma	113 (88.3%)	46 (69.7%)	0.001	109 (82.6%)	50 (80.6%)	0.744	29 (72.5)	130 (84.4)	0.081	128 (85.3)	31 (70.5)	0.024
	Non Endometrioid adenocarcinoma	15 (11.7%)	20 (30.3%)		23 (17.4%)	12 (19.4%)		11 (27.5%)	24 (15.6%)		22 (14.7%)	13 (29.5%)	
Differentiation grading	G1	49 (38.3%)	25 (37.9%)	0.773	51 (38.6%)	23 (37.1%)	0.604	16 (40.0%)	58 (37.7%)	0.892	59 (39.3%)	15 (34.1%)	0.776
	G2	57 (44.5%)	27 (40.9%)		59 (44.7%)	25 (40.3%)		16 (40.0%)	68 (44.2%)		63 (42.0%)	21 (47.7%)	
	G3	22 (17.2%)	14 (21.2%)		22 (16.7%)	14 (22.6%)		8 (20.0%)	28 (18.2%)		28 (18.7%)	8 (18.2%)	
Largest diameter of tumor (cm)	< 2	37 (28.9%)	14 (21.2%)	0.249	32 (24.2%)	19 (30.6%)	0.345	8 (20.0%)	43 (27.9%)	0.311	45 (30.0%)	6 (13.6%)	0.03
	≥ 2	91 (71.1%)	52 (78.8%)		100 (75.8%)	43 (69.4%)		32 (80.0%)	111 (72.1%)		105 (70.0%)	38 (86.4%)	
Myometrial invasion depth	< 1/2	99 (77.3%)	44 (66.7%)	0.109	96 (72.7%)	47 (75.8%)	0.65	29 (72.5%)	114 (74.0%)	0.845	117 (78.0%)	26 (59.1%)	0.012
	≥ 1/2	29 (22.7%)	22 (33.3%)		36 (27.3%)	15 (24.2%)		11 (27.5%)	40 (26.0%)		33 (22.0%)	18 (40.9%)	
Cervical stromal involvement	No	106 (82.8%)	56 (84.8%)	0.717	113 (85.6%)	49 (79.0%)	0.25	30 (75.0%)	132 (85.7%)	0.104	127 (84.7%)	35 (79.5%)	0.421
	Yes	22 (17.2%)	10 (15.2%)		19 (14.4%)	13 (21.0%)		10 (25.0%)	22 (14.3%)		23 (15.3%)	9 (20.5%)	
Lymph node metastasis	No	111 (86.7%)	56 (84.8%)	0.721	119 (90.2%)	48 (77.4%)	0.017	37 (92.5%)	130 (84.4%)	0.188	130 (86.7%)	37 (84.1%)	0.664
	Yes	17 (13.3%)	10 (15.2%)		13 (9.8%)	14 (22.6%)		3 (7.5%)	24 (15.6%)		20 (13.3%)	7 (15.9%)	
Postoperative adjuvant therapy	No	52 (40.6%)	22 (33.3%)	0.322	52 (39.4%)	22 (35.5%)	0.601	9 (22.5%)	65 (42.2%)	0.022	63 (42.0%)	11 (25.0%)	0.041
	Yes	76 (59.4%)	44 (66.7%)		80 (60.6%)	40 (64.5%)		31 (77.5%)	89 (57.9%)		87 (58.0%)	33 (75.0%)	

Abbreviations: BMI: body mass index; EC: endometrial cancer; FIGO: International Federation of Obstetrics and Gynecology; HDL‐C: high‐density lipoprotein cholesterol; LDL‐C: low‐density lipoprotein cholesterol; TC: total cholesterol; TG: triglycerides.

Regarding the cause of death, 18 patients died from EC, five from cardiovascular or cerebrovascular diseases, and eight from other causes. A univariate Cox proportional hazards analysis was conducted to evaluate the association between the OS of patients with EC and various clinical characteristics, as summarized in Table 1. The results showed that age at diagnosis (HR = 1.068; 95% CI: 1.014–1.102; *p* = 0.009), TG ≥ 1.7 mmol/L (HR = 2.870; 95% CI: 1.414–5.826; *p* = 0.004), HDL‐C < 1.0 mmol/L (HR = 4.156; 95% CI: 2.053–8.415; *p* = 0.001), FIGO stage II or III (HR = 4.656; 95% CI: 2.300–9.428; *p* = 0.001), and non‐endometrioid adenocarcinoma (HR = 3.964; 95% CI: 1.940–8.099; *p* = 0.001) were risk factors affecting the OS of patients with EC. However, menopausal status, BMI, TC, LDL‐C, tumor differentiation grading, myometrial invasion depth, cervical stromal involvement, and lymph node metastasis were not associated with OS in patients with EC (Table 2).

**TABLE 2:** Univariate Cox proportional hazards analysis of OS in patients with EC.

Variables	*β*	Standard error	Wald	*p*	HR	95% confidence interval	
Age at diagnosis (years)	0.065	0.022	8.535	0.003	1.068	1.022	1.115
Postmenopausal (Yes vs. No)	0.375	0.369	1.036	0.309	1.456	0.707	2.999
BMI (≥ 25 kg/m^2^ vs. < 25 kg/m^2^)	−0.039	0.360	0.012	0.913	0.961	0.475	1.945
TC (≥ 5.2 mmol/L vs. < 5.2 mmol/L)	0.467	0.361	1.675	0.196	1.595	0.786	3.237
TG (≥ 1.7 mmol/L vs. < 1.7 mmol/L)	1.054	0.361	8.524	0.004	2.870	1.414	5.826
LDL‐C (≥ 3.4 mmol/L vs. < 3.4 mmol/L)	0.521	0.384	1.837	0.175	1.684	0.793	3.577
HDL‐C (< 1.0 mmol/L vs. ≥ 1.0 mmol/L)	1.425	0.360	15.665	0.001	4.156	2.053	8.415
FIGO stage (II or III vs. < I)	1.538	0.360	18.262	0.001	4.656	2.300	9.428
Pathological type (Non Endometrioid adenocarcinoma vs. Endometrioid adenocarcinoma)	1.377	0.365	14.275	0.001	3.964	1.940	8.099
Differentiation grading (ref. G3)	0.000				1.000		
Differentiation grading (G2)	−0.446	0.403	1.226	0.268	0.640	0.291	1.410
Differentiation grading (G1)	−0.206	0.488	0.178	0.673	0.814	0.313	2.118
Lagest diameter of tumor (≥ 2 cm vs. < 2 cm)	0.640	0.488	1.718	0.190	1.897	0.728	4.940
Myometrial invasion depth (≥ 1/2 vs. < 1/2)	0.483	0.375	1.652	0.199	1.620	0.776	3.383
Cervical stromal involvement (Yes vs. No)	0.518	0.430	1.456	0.228	1.679	0.723	3.899
Lymph node metastasis (Yes vs. No)	0.674	0.430	2.464	0.116	1.963	0.846	4.557
Postoperative adjuvant therapy (Yes vs. No)	0.632	0.410	2.368	0.124	1.881	0.841	4.205

Abbreviations: BMI: body mass index; EC: endometrial cancer; FIGO: International Federation of Obstetrics and Gynecology; HDL‐C: high‐density lipoprotein cholesterol; HR: hazard ratio; LDL‐C: low‐density lipoprotein cholesterol; OS: overall survival; TC: total cholesterol; TG: triglycerides.

Furthermore, the results of the multivariate Cox proportional hazards analysis showed that the OS of patients with EC was significantly decreased in the TG ≥ 1.7 mmol/L and HDL‐C < 1.0 mmol/L groups (HR = 2.631, 95% CI: 1.260–5.495, *p* = 0.010; HR = 2.423, 95% CI: 1.144–5.133, *p* = 0.021, respectively, Table 3). Meanwhile, the OS of patients with EC was significantly decreased in older patients (HR = 1.057, 95% CI: 1.014–1.102, *p* = 0.009), the advanced FIGO stage (II or III) group (HR = 3.735, 95% CI: 1.755–7.941, *p* < 0.001), and the non‐endometrioid adenocarcinoma group (HR = 2.744, 95% CI: 1.293–5.825, *p* = 0.009, Table 3).

**TABLE 3:** Multivariate Cox proportional hazards analysis of OS in patients with EC.

Variables	*β*	Standard error	Wald	*p*	HR	95% confidence interval	
Age at diagnosis (years)	0.055	0.021	6.812	0.009	1.057	1.014	1.102
TG (≥ 1.7 mmol/L vs. < 1.7 mmol/L)	0.967	0.376	6.627	0.010	2.631	1.260	5.495
HDL‐C (< 1.0 mmol/L vs. ≥ 1.0 mmol/L)	0.885	0.383	5.343	0.021	2.423	1.144	5.133
FIGO stage (II or III vs. I)	1.318	0.385	11.694	< 0.001	3.735	1.755	7.947
Pathological type (Non Endometrioid adenocarcinoma vs. Endometrioid adenocarcinoma)	1.009	0.384	6.909	0.009	2.744	1.293	5.825

Abbreviations: BMI: body mass index; EC: endometrial cancer; FIGO: International Federation of Obstetrics and Gynecology; HDL‐C: high‐density lipoprotein cholesterol; OS: overall survival; TG: triglycerides.

**FIGURE 2:**
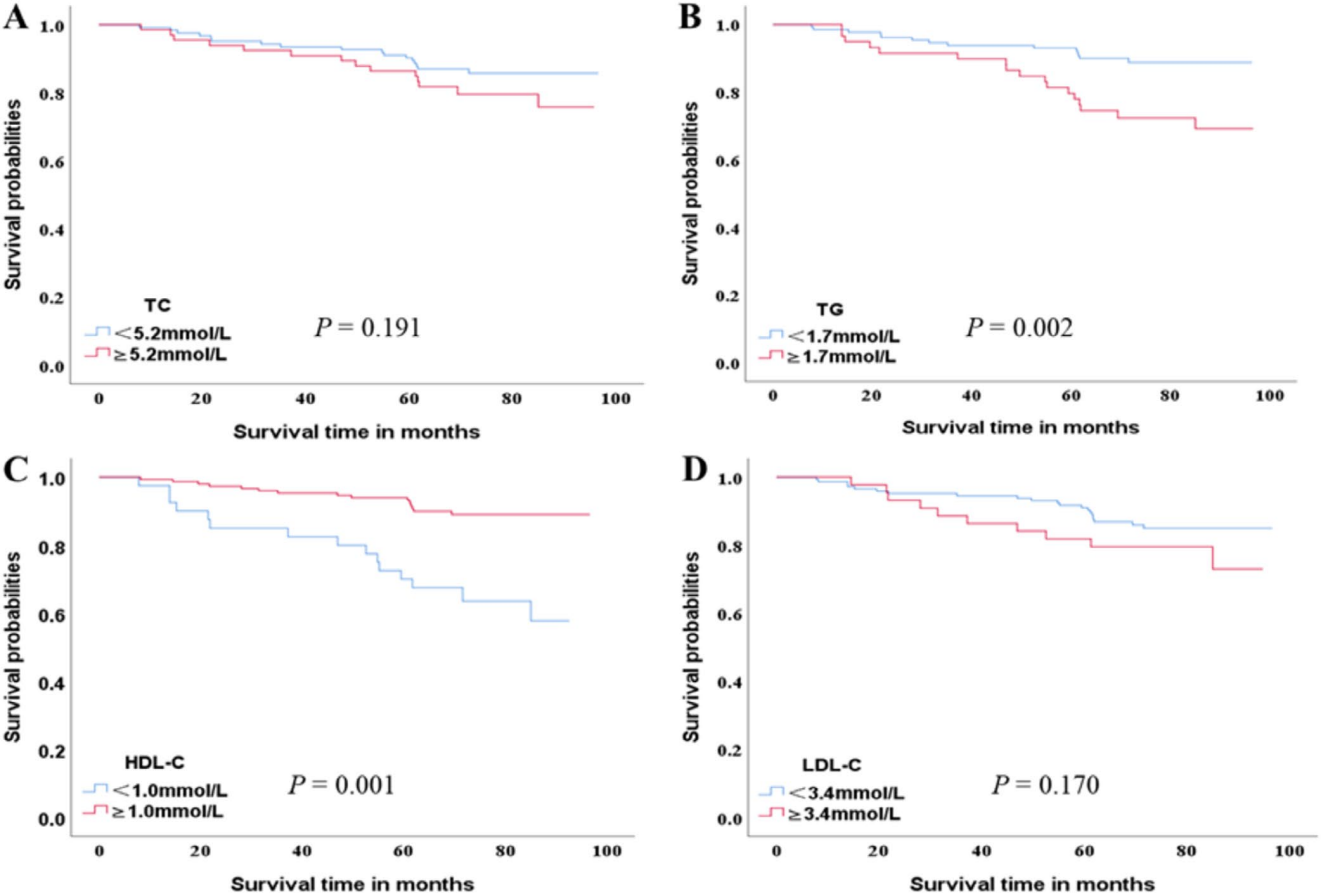
Survival curves via Kaplan–Meier analysis of different levels of TC (A), TG (B), HDL‐C (C), and LDL‐C (D). HDL‐C: high‐density lipoprotein cholesterol; LDL‐C: low‐density lipoprotein cholesterol; TC: total cholesterol; TG: triglycerides.

Log rank tests were used to analyze the survival status of patients with EC in low‐level and high‐level lipid groups, as shown in Table 4. The results showed that the OS rate was significantly different between the two groups (*p* < 0.05). Kaplan–Meier curves of the survival status of patients with different lipid levels are shown in Figure 2.

**TABLE 4:** The log‐rank test of OS in EC patients with different lipids levels.

		Median survival time (month)	Standard error	95% confidence interval		Log‐rank χ^2^	*p*
TC	< 5.2 mmol/L	88.9	1.870	85.261	92.591	1.706	0.191
	≥ 5.2 mmol/L	84.4	2.953	78.581	90.155		
TG	< 1.7 mmol/L	90.2	1.706	86.810	93.497	9.344	0.002
	≥ 1.7 mmol/L	81.6	3.375	75.000	88.232		
HDL‐C	< 1.0 mmol/L	72.8	4.539	63.935	81.729	18.488	0.001
	≥ 1.0 mmol/L	90.9	1.462	88.037	93.767		
LDL‐C	< 3.4 mmol/L	88.8	1.719	85.469	92.207	1.880	0.170
	≥ 3.4 mmol/L	82.0	3.778	74.549	89.361		

Abbreviations: EC: endometrial cancer; HDL‐C: high‐density lipoprotein cholesterol; LDL‐C: low‐density lipoprotein cholesterol; OS: overall survival; TC: total cholesterol; TG: triglycerides.

## 4 Discussion

Our retrospective study showed that preoperative dyslipidemia was related to the FIGO stage, pathological type, myometrial invasion depth, and lymph node metastasis of EC. Particularly, high‐level TG (≥ 1.7 mmol/L) and low‐level HDL‐C (< 1.0 mmol/L) levels were closely associated with the reduced OS in patients with EC, indicating that preoperative dyslipidemia could serve as important biomarkers for evaluating the prognosis of patients with EC.

Metabolic disorders play an important role in the development and progression of malignant tumors. Metabolic syndrome is associated with a higher risk and poorer OS of EC [16, 17]. Dyslipidemia, one of the important features of metabolic syndrome, has also been reported as a risk factor for EC. Findings from a meta‐analysis of observational studies support the positive association between dietary cholesterol consumption and the risk of EC [18]. A study by Luo et al. on 631 postmenopausal women found that a TG/HDL‐C ratio ≥ 1.52 is an independent predictor of EC [15]. The TG/HDL‐C ratio increased significantly with advanced tumor stage and was positively associated with non‐endometrioid adenocarcinoma [19]. Moreover, HDL‐C was associated with an increased risk of EC‐related death in the multivariate‐adjusted model, after adjusting for age, histological type, tumor grade, and stage [20]. However, these previous studies did not exclude patients with hypertension or diabetes, which could confound their conclusions. In our present study, we have found that preoperative dyslipidemia was correlated with some clinical characteristics and the OS of patients with EC after excluding those with hypertension and diabetes, suggesting that abnormal blood lipid metabolism before progressing to organ or system dysfunction might relate to the prognosis of EC, consistent with the findings of previous studies.

Our data showed that the proportions of non‐endometrioid adenocarcinoma, FIGO stage greater than I, largest tumor diameter ≥ 2 cm, myometrial invasion depth ≥ half, and lymph node metastasis were higher in patients with dyslipidemia, which might infer a worse prognosis in these patients. Previous studies reveal similar results. Shi et al. found that the triglyceride‐glucose index was positively associated with advanced pathological stage (OR: 2.14, 95% CI: 1.32–3.47, *p* = 0.002) and poorer differentiation (OR: 2.53, 95% CI: 1.36–4.72, *p* = 0.004) [21], indicating the risk of EC incidence and progression. Additionally, the metabolic‐inflammatory‐nutritional score developed by Lin et al. could predict lymph node metastasis (LNM) in EC [22]. Therefore, clinicians should be cautious about the necessity of extended surgery and closer postoperative follow‐up in patients with preoperative dyslipidemia.

The study by Luo et al. reported the relationship between the TG/HDL‐C ratio and clinicopathological characteristics in 167 patients with EC but did not examine their survival [15]. High TG levels have been reported to have a negative impact on the OS of patients with colorectal cancer, non‐small cell lung cancer, and breast cancer [9, 10, 11]. However, its influence on the prognosis of patients with EC is unknown. In the present study, we demonstrate for the first time that preoperative hypertriglyceridemia or low HDL‐C correlated with the reduced survival time of patients with EC, consistent with the findings of previous studies on other malignancies. We conducted telephone follow‐ups to track the cause of patients' deaths. However, because the cause of death was mainly obtained through a telephone inquiry from family members, it may be inaccurate. Therefore, we only calculated the all‐cause mortality in our study. A study analyzing population‐based EC data from the National Cancer Institute's Surveillance, Epidemiology, and End Results Program in the United States found that patients with EC were most likely to die from EC, followed by cardiovascular disease, other cancers, and chronic obstructive pulmonary disease [23]. The patterns and trends in the cause of death among the patients in our study require further research.

We observed that the proportion of those undergoing postoperative adjuvant therapy was higher among patients with dyslipidemia, especially among those in the low HDL‐C and high LDL‐C groups. This may be due to the poorer tumor characteristics in this population, because more patients with dyslipidemia have an advanced pathological stage, non‐endometrioid adenocarcinoma, the largest diameter of tumor ≥ 2 cm, myometrial invasion depth ≥ 1/2, cervical stromal involvement, and LNM. Moreover, the proportion of patients who underwent postoperative adjuvant therapy was higher among those with dyslipidemia, which might partly explain why a high level of TC or LDL‐C was not associated with the OS of patients with EC.

The detailed mechanism of how dyslipidemia influences the prognosis of EC remains unknown. Lipid metabolism reprogramming and aberrant transcriptional regulation of the lipid metabolism pathway have been recognized as the hallmark of EC [24]. A study by Luo et al. [15] suggested that high TG levels could increase the volume and number of adipocytes, which produce aromatase, an enzyme that converts androstenedione to estrogen, providing estrogen in postmenopausal women and inducing the continuous proliferation of endometrial cells. Lack of antagonism to estrogen from progesterone could ultimately lead to uncontrolled cell proliferation, increasing the risk of EC. Moreover, high lipid levels would accelerate LNM of EC, as lipids need to be transferred to the chyle pool through the lymphatic system. Additionally, dyslipidemia induces sustained production of inflammatory cytokines and leads to chronic inflammation, which ultimately results in the inhibition of anti‐tumor immune cells [11].

As dyslipidemia might affect the survival of EC, the use of statins may improve the prognosis of patients with EC and dyslipidemia. Several studies have shown the impact of statin treatment on improving the survival of patients with EC. Based on the Danish Cancer Registry, it was reported that post‐diagnosis statin use was associated with a lower mortality rate compared to non‐statin use among 6694 women with EC [25]. A study by Li et al., who analyzed the data of 5923 women with EC from five countries, showed that the use of statins prolonged both OS and disease‐specific survival [26]. In contrast, a group of Israeli researchers reported that the 5‐year relapse‐free survival and the OS were similar between patients with EC who used statins before diagnosis and those who had not used them at all [27]. Although the impact of statin treatment on the course of EC appears inconclusive, the proportion of results suggesting a beneficial effect of statins on EC progression seems promising.

This study has several limitations, including a small sample size, a retrospective design, selection bias, and the lack of monitoring and control of postoperative blood lipid levels. Regarding the timing of lipid measurements, it might be difficult to determine causality between dyslipidemia and the risk of EC. For example, the low HDL‐C might be a result of the disease burden. Study from Hu et al. had shown that progestin‐resistant patients with EC exhibited reduced serum apolipoprotein A‐I concentrations and increased cholesterol accumulation in endometrial tissue [28], which might partly explain the low HDL‐C level in patients with progressive EC. However, in our present study, we have paid close attention to the prognosis of patients with EC who were diagnosed with preoperative dyslipidemia, especially the OS. Because these patients were followed up for a couple of years, during which death occurred after the appearance of dyslipidemia, our result is reliable to a certain extent. Moreover, the multivariate Cox proportional hazards analysis strengthens the reliability of our findings. We explicitly acknowledge that our cases were drawn from those without the appearance of hypertension, diabetes and coronary heart disease, which may affect the direct applicability of the results to broader populations. In view of this, we acknowledge that the findings of our research are more intended as exploratory or biomarker‐oriented analysis rather than a generalizable prognostic model.

The included cases in our manuscript were diagnosed and treated between June 2013 and December 2016, which could ensure relatively sufficient follow‐up time. Patients with EC during this period were staged with FIGO 2009 criteria, which is a classic staging system and has been used for a long time. The use of the older staging system was due to the included cases and follow‐up time needed in our study. This staging system was updated in 2023 by FIGO, which added histology, LVSI, and molecular classification. Moreover, the ESGO/ESMO risk stratification and molecular classification have been widely applied in clinical practice worldwide nowadays. Therefore, the applicability of the findings in our study might be limited in the current staging system and contemporary risk stratification of EC. Nevertheless, it was applicable to some patients with EC in the development district, of which molecular classification has not been extensively promoted. More prospective studies with larger sample sizes are required to gain a clearer understanding of the impact of blood lipid levels on the survival of patients with EC.

In conclusion, we found that preoperative TG and HDL‐C were associated with the reduced OS of EC, suggesting the need for aggressive surgical treatments and close follow‐up of patients with EC and dyslipidemia to improve their prognosis.

## Funding

This work was supported by the National Key Research and Development Program, 2024YFC2707503; Sanming Project of Medicine in Shenzhen, SZSM202211043; Sanming Project of Cervical Disease Prevention and Control from Pingshan District Maternal and Child Health Hospital and Shenzhen Peking University Shenzhen Hospital, 2023‐1; and Shenzhen Science and Technology Program, RCBS20231211090710015.

## Conflicts of Interest

The authors declare no conflicts of interest.

## Supporting information


Table S1.

## Data Availability

The data that support the findings of this study are available on request from the corresponding author. The data are not publicly available due to privacy or ethical restrictions.
