# Supplementary material for: Association Between Preoperative Dyslipidemia and the Prognosis of Patients With Endometrial Cancer: A Retrospective Cohort Study
Source: Kaohsiung J Med Sci. 2026 Mar 9:e70195. Online ahead of print. doi: 10.1002/kjm2.70195 (PMC13399785; doi:10.1002/kjm2.70195)
Supplement: Supplementary file 1 — Table S1. [file KJM2-9999-e70195-s001.docx]

**Table S1 The basic characteristics of included patients with EC**

| Clinical and pathological characteristics | | n (%) |  |
| --- | --- | --- | --- |
|  |  |  |  |
| Age at diagnosis (years) | < 55 | 121(62.4) |  |
|  | ≥ 55 | 73(37.6) |  |
| Menopause status | Premenopausal | 93(47.9) |  |
|  | Postmenopausal | 101(52.1) |  |
| BMI (kg/m^2^) | < 25 | 92(47.4) |  |
|  | ≥ 25 | 102(52.6) |  |
| TC (mmol/L) | < 5.2 | 128(66) |  |
|  | ≥ 5.2 | 66(34) |  |
| TG (mmol/L) | < 1.7 | 132(68) |  |
|  | ≥ 1.7 | 62(32) |  |
| LDL-C (mmol/L) | < 3.4 | 150(77.3) |  |
|  | ≥ 3.4 | 44(22.7) |  |
| HDL-C (mmol/L) | < 1.0 | 40(20.6) |  |
|  | ≥ 1.0 | 154(79.4) |  |
| FIGO stage | I | 156(80.4) |  |
|  | >I (II+III) | 38(19.6) |  |
| Pathological type | Endometrioid adenocarcinoma | 159(82) |  |
|  | Non Endometrioid adenocarcinoma | 35(18) |  |
| Differentiation grading | G1 | 74(38.1) |  |
|  | G2 | 84(43.3) |  |
|  | G3 | 36(18.6) |  |
| Lagest diameter of tumor (cm) | < 2 | 51(26.3) |  |
|  | ≥ 2 | 143(73.7) |  |
| Myometrial invasion depth | < 1/2 | 143(73.7) |  |
|  | ≥ 1/2 | 51(26.3) |  |
| Cervical stromal involvement | No | 162(83.5) |  |
|  | Yes | 32(16.5) |  |
| Lymph node metastasis | No | 167(86.1) |  |
|  | Yes | 27(13.9) |  |
| Survival status | Survival | 163(84) |  |
|  | Dead | 31(16) |  |
| Postoperative adjuvant therapy (Yes vs No) | Yes | 120(61.9) |  |
|  | No | 74(38.1) |  |

EC: endometrial cancer; BMI: body mass index; TC: total cholesterol; TG: triglycerides; HDL-C: high-density lipoprotein cholesterol; LDL-C: low-density lipoprotein cholesterol; FIGO: International Federation of Obstetrics and Gynecology.
